# Elusive data underlying debate at the prokaryote-eukaryote divide

**DOI:** 10.1186/s13062-018-0221-x

**Published:** 2018-10-03

**Authors:** Marie Gerlitz, Michael Knopp, Nils Kapust, Joana C. Xavier, William F. Martin

**Affiliations:** Institute for Molecular Evolution, Heinrich-Heine-University Düsseldorf, Universitätsstr. 1, 40225 Düsseldorf, Germany

**Keywords:** Eukaryogenesis, Mitochondria, Ribosomes, Bioenergetics, Major evolutionary transitions

## Abstract

**Background:**

The origin of eukaryotic cells was an important transition in evolution. The factors underlying the origin and evolutionary success of the eukaryote lineage are still discussed. One camp argues that mitochondria were essential for eukaryote origin because of the unique configuration of internalized bioenergetic membranes that they conferred to the common ancestor of all known eukaryotic lineages. A recent paper by Lynch and Marinov concluded that mitochondria were energetically irrelevant to eukaryote origin, a conclusion based on analyses of previously published numbers of various molecules and ribosomes per cell and cell volumes as a presumed proxy for the role of mitochondria in evolution. Their numbers were purportedly extracted from the literature.

**Results:**

We have examined the numbers upon which the recent study was based. We report that for a sample of 80 numbers that were purportedly extracted from the literature and that underlie key inferences of the recent study, more than 50% of the values do not exist in the cited papers to which the numbers are attributed. The published result cannot be independently reproduced. Other numbers that the recent study reports differ inexplicably from those in the literature to which they are ascribed. We list the discrepancies between the recently published numbers and the purported literature sources of those numbers in a head to head manner so that the discrepancies are readily evident, although the source of error underlying the discrepancies remains obscure.

**Conclusion:**

The data purportedly supporting the view that mitochondria had no impact upon eukaryotic evolution data exhibits notable irregularities. The paper in question evokes the impression that the published numbers are of up to seven significant digit accuracy, when in fact more than half the numbers are nowhere to be found in the literature to which they are attributed. Though the reasons for the discrepancies are unknown, it is important to air these issues, lest the prominent paper in question become a point source of a snowballing error through the literature or become interpreted as a form of evidence that mitochondria were irrelevant to eukaryote evolution.

**Reviewers:**

This article was reviewed by Eric Bapteste, Jianzhi Zhang and Martin Lercher.

## Background

Lynch and Marinov [1] recently tabulated published values for the numbers of various molecules in cells in relationship to the cell volume with the aim of investigating the prokaryote to eukaryote transition. In the main, Lynch and Marinov [1] concluded from their calculations that “*there is no reason to think membrane bioenergetics played a direct, causal role in the transition from prokaryotes to eukaryotes and the subsequent explosive diversification of cellular and organismal complexity*”. Their arguments claiming the evolutionary insignificance of mitochondria have been countered elsewhere [2], here the issue concerns irregularities in their published data. The keystone of their paper is a seemingly impressive list of values for the volumes of cells, their surface area, numbers of ATPases, and numbers of ribosomes per cell, numbers that are carefully tabulated in the supplementary information together with the corresponding references that serve as the paper’s foundation. Their paper and its underpinning supplementary information appear to be a rich source of useful numbers for calculations about various processes in cells, provided that the numbers are accurate. We checked.

## Main text

The thrust of Lynch and Marinov’s [1] paper is a specific critique of a view attributable to one of us (WFM), namely that mitochondria were essential to the prokaryote-eukaryote transition [3, 4], therefore it is fair to inspect the strength of the data upon which the criticisms rest. In peer review, no one seems to have questioned whether their numbers were accurate. As we read Lynch and Marinov [1], we noticed that some of the numbers in Appendix 1-Tables 1–3 were conspicuously precise, often carrying up to eight significant digits for estimates of the numbers of ribosomes per cell or the volume of a cell in μm^3^. Do such values exist in the literature? Upon checking, we found that some form of systematic error underlies the paper by Lynch and Marinov [1].

Initially our attention was drawn to the number of cytosolic ribosomes for *Tetrahymena pyriformis*, reported as 7,490,000 [1] (Appendix 1-Table 3), which seemed very different from the value for *Chlamydomonas reinhardtii*. We consulted Hallberg and Bruns [5] to which the value of 7,490,000 ribosomes per cell was attributed, but the only number in that paper containing the sequence of digits 7.49 is on page 385, Table 1, column 2, row 13; the number is not the number of ribosomes per cell, it is the value of an optical density measured at 259 nm (OD_259_) per million *Tetrahymena* cells. That spotcheck prompted further checks and it soon became apparent that a complete check of all the numbers in their Appendix 1-Table 3 was warranted. Appendix 1-Table 3 underlies Fig. 2 of Lynch and Marinov [1], which plotted cell volume against the number of ribosomes per cell and contained data points for 26 out of 27 species listed in Appendix 1-Table 3 (one of the species was not plotted, for unknown reasons). For two species, *Vibrio angustum* and *Glycine max* SB-1 cell, no values for cell volume were provided in Appendix 1-Table 3. All the other values in this table were checked in the original references and the results of this exercise are summarized in Tables 1 and 2.

**Table 1:** Analysis of cell volumes and respective cited literature provided in Lynch and Marinov [1]

*Species*	Volume (μm^3^)	Reference(s) cited	Location in cited reference^1,2^	Comments
*Bacillus subtillis*	1.407	Barrera and Pan [14]	not present	The value reported is a range: 0.85-1.13 μm^3^ (p. S18, Table S-3, col. 4-5, rows 3-5).
		Maass et al. [15]	not present	
*Escherichia coli*	0.983	Bremer and Dennis [16]	not present	
		Fegatella et al. [17]	not present	The value reported is 1.1 μm^3^ (p. 4434, col. 2, l. 30).
		Bakshi et al. [18]	not present	The value reported is a range from 1.3-2.4 μm^3^ (p. S12, Fig S6).
		Arfvidsson and Wahlund [19]	not present	
		Wisniewski et al. [20]	not present	
		Lu et al. [21]	not present	
*Legionella pneumophila*	0.58	Leskelä et al. [22]	not present	
*Leptospira interrogans*	0.22	Beck et al. [23]	p. S10, col 1, l. 8	
		Schmidt et al. [24]	not present	
*Mycoplasma pneumoniae*	0.05	Yus et al. [25]	not present	The value in Maier et al. is from Hasselbring [29]. Still not plotted at 0.05 μm^3^ in Figure 2.
		Seybert et al. [26]	not present	
		Kühner et al. [27]	not present	
		Maier et al. [28]	p. S3, col. 1, l. 5	
*Mycobacterium tuberculosis*	0.215	Yamada et al. [30]	not present	
*Rickettsia prowazekii*	0.089	Pang and Winkler [31]	not present	The value reported is close but not the same: 0.09 μm^3^ (p. 117, Table 2, comment e.).
*Sphingopyxis alaskensis*	0.05	Fegatella et al. [17]	p. 4434, col. 2, l. 29	
*Spiroplasma melliferum*	0.018	Ortiz et al. [32]	incorrect	L&M take the volume for a portion of the cell (p.339, col .1, l. 10). The volume reported for the whole cell is 34 times larger (p.339, col. 2, l. 16-18).
*Staphylococcus aureus*	0.288	Martin and Iandolo [33]	not present	
*Vibrio angustum*	no value	Flärdh et al. [34]		
ARMAN*	0.03	Comolli et al. [35]	p. 162, col. 2, l. 41	
Exophiala dermatitidis*	43.8	Biswas et al. [36]	p. 137, Table 2, col. 7, row 2, 4	L&M add the reported mean numbers for cell (36.0 ± 12.6 μm^3^) and cell wall (7.8 ± 2.5 μm^3^).
*Saccharomyces cerevisiae*	69.071	Warner [37]	not present	The reported values are 17.1 μm^3^ for a cell in G1 and 13.3 μm^3^ for a cell in the early G1 (p.326, Table 2, l. 3).
		Yamaguchi et al [38]	not present	
		Kulak et al. [39]	not present	
		Ghaemmaghami et al. [40]	not present	
*Schizosaccharomyces pombe*	118	Marguerat et al. [41]	not present	The reported values are: cell length of 5-15 μm and cell diameter of 3.5 μm. p.322, col. 2, l. 24.
		Maclean [42]	not present	
		Kulak [39]	not present	
*Tetrahymena pyriformis*	14002.067	Hallberg and Bruns [5]	not present	
*Tetrahymena thermophila*	7856.00	Calzone et al. [43]	not present	
*Chlamydomonas reinhardtii*	151.00	Bourque et al. [44]	not present	
Ostreococcus tauri*	0.910	Henderson et al. [45]	p. 3, col. 1, l. 7	
*Adonis aestivalis* (vegetative)	2380.3	Lin and Gifford [46]	p. 2481, Table 2, col. 3, row 3-5	L&M take the mean value for the vegetative apex of the central zone (2877), peripheral zone (924) and rib meristem (3340).
*Adonis aestivalis* (transitional)	2287.00	Lin and Gifford [46]	p. 2481, Table 2, col. 3, row 6-8	L&M take the mean value for the vegetative apex of the transitional zone (1721), peripheral zone (1095) and rib meristem (4045).
*Adonis aestivalis*	2690.00	Lin and Gifford [46]	p. 2481, Table 2 col. 3, row 9-11	L&M take the mean value for the vegetative apex of the floral zone (2380), peripheral zone (802) and rib meristem (4888).
Glycine max SB-1 cell	no value	Jackson and Lark [47]		
Rhus toxicodendron*	1222.00	Vassilyev [48]	p.617, Table 2, col 2-3, row 1	L&M take the mean value of the whole cell for procambial stage (1028), rough ER stage (1130) and smooth ER stage (1508).
*Zea mays* root cell	240,000	Hsiao [49]	not present	The value appears in the literature but not in Hsiao [49], but rather in Hsiao [50] p.105, Table 1, col. 3, row 4-5.
Hamster, intestinal enterocyte*	1890.00	Buschmann and Manke [51, 52]	p. 16, Table 1, col. 2, row 6	In Buschmann and Manke [52].
HeLa Cell	2798.668	Duncan and Hershey [53]	not present	The reported value is 2600 μm^3^. p.163, col. 2, l. 35.
		Zhao et al. [54]	not present	
		Kulak et al. [39]	not present	
Mouse pancreas*	1434.00	Dean [55]	p. 117, Table 2, col. 6, row 2	
Rat liver cell*	4940.00	Weibel et al. [56]	p. 80, Table 2, col. 10, row 2	

^1^‘S’ refers to Supplemental Material

^2^not present - value cannot be found in the cited paper; incorrect - a wrong value was taken from the cited paper

*Species with both volume and ribosome count values verified

**Table 2:** Analysis of ribosome numbers and respective cited literature provided in Lynch and Marinov [1]

Species	Ribosome number	Reference(s) cited	Location in cited reference^1,2^	Comments
*Bacillus subtilis*	6000	Barrera and Pan [14]	p. 485, col. 1, l. 30	
	9124	Maass et al. [15]	incorrect	L&M take the average of the quantification for the four ribosomal proteins for which there is data in this study, in all three time points. However, rplL should be scaled down as it is present in 4 copies per ribosome^3^.
*Escherichia coli*	72000	Bremer and Dennis [16]	p. 9, Table 3, col. 8, row 24	
	45100	Fegatella et al. [17]	p. 4437, Table 3, col. 4, row 9	
	26300	Fegatella et al. [17]	p. 4437, Table 3, col. 4, row 8	
	13500	Fegatella et al. [17]	p. 4437, Table 3, col. 4, row 7	
	6800	Fegatella et al. [17]	p. 4437, Table 3, col. 4, row 6	
	55000	Bakshi et al. [18]	p. 26, col. 2, l. 13	
	20100	no reference provided		
	12000	Arfvidsson and Wahlund [19]	not present	L&M seem to have extrapolated the value from Figure 5, p. 82, however the figure shows a range of ca. 2500-13000.
	6514	Wiśniewski et al. [20]	not present	The citation seems to be incorrect. No reference to *E. coli* in the citation.
	17979	Lu et al. [21]	incorrect	L&M take the average of the quantification of all rpl and rps proteins but exclude rpm proteins. Also, rpl should be scaled down as it is present in 4 copies per ribosome^3^.
*Legionella pneumophila*	7400	Leskelä et al. [22]	p. 174, col. 2, l. 24	
*Leptospira interrogans*	4500	Beck et al. [23]	p. 820, Table 1, col. 2, l. 3	L&M use 4500, which is one number in the range reported: 3400, 3500, 4500. Considering standard deviations, the range reported is 2800-5000.
	1039	Schmidt et al. [24]	incorrect	L&M take the average of the quantification of all ribosomal proteins for the first time-point of the serum treatment (same value for the doxycycline treatment). The range of averages for all time points and all treatments is 537-2170. The range for the serum treatment is 537-1039.
*Mycoplasma pneumoniae*	140	Yus et al. [25]	p. S14, col. 1, l. 23	Yus et al. cite their value from “Kuhner et al. accompanying manuscript”.
	300	Seybert et al. [26]	p. 351, col. 1, l. 39	
	422	Kühner et al. [27]	p. S38, Figure S9B, l. 9	L&M use the values of the Western Blot estimation. The electron tomography results of the same citation indicate 140
	225	Maier et al. [28]	incorrect	L&M take the mean of all values in Table S7 column “direct quantified (copies per cell)”. However, RPL7 needs to be scaled down as it is present more than once per ribosome^3^.
*Mycobacterium tuberculosis*	1672	Yamada et al. [30]	p. 9, Table 4, col. 4, row 7	
*Rickettsia prowazekii*	1500	Pang and Winkler [31]	p. 117, Table 2, col. 2, row 6	
*Sphingopyxis alaskensis*	1850	Fegatella et al. [17]	not present	
	200	Fegatella et al. [17]	p. 4435, col. 1, l. 27	The value reported is 2000 (p. 4435, col. 1, l. 24).
*Spiroplasma melliferum*	275	Ortiz et al. [32]	incorrect	L&M take the number of ribosomes for a portion of the cell. The number reported for the whole cell is 1000 (p.339, col. 2, l. 20).
*Staphylococcus aureus*	54400	Martin and Iandolo [33]	p. 1139, Table 1, col. 2-3, l. 9	L&M take the mean of estimated ribosomes per cell in rich and poor medium: 83200 (col. 2, row 11); 25600 (col. 3, row 11).
Vibrio angustum	27500	Flärdh et al. [34]	p. 6783, col. 1, l. 13	L&M take the mean of two counts: 20000 and 35,000.
	8000	Flärdh et al. [34]	p. 6783, col. 1, l. 14	The reported value is after 4 days of starvation. Value for 24h of starvation (16000) not included.
ARMAN*	92	Comolli et al. [35]	p. 162, col. 2, l. 41	
Exophiala dermatitidis*	195000	Biswas et al. [36]	p. 137, Table 1, col. 7, row 6	
*Saccharomyces cerevisiae*	200000	Warner [37]	p. 437, col. 1, l. 34	
	220000	Yamaguchi et al. [38]	incorrect	L&M take the value from the abstract, which is an approximation. The reported value is 217000 (mean for G1 cells - p.325, Table 1, col. 5, row 11).
	153456	Kulak et al. [39]	not reproducible	Not clear how the value was calculated.
	72284	Ghaemmaghami et al. [40]	not reproducible	Not clear how the value was calculated.
*Schizosaccharomyces pombe*	150000	Marguerat et al. [41]	p. 677, col. 1, l. 35	
	500000	Maclean [42]	p. 323, col. 1, l. 66	
	505260	Kulak [39]	not reproducible	Not clear how the value was calculated.
	100568	Marguerat et al. [41]	not reproducible	Not clear how the value was calculated.
*Tetrahymena pyriformis*	7490000	Hallberg and Bruns [5]	incorrect	L&M take the reported number from p. 385, table 1, col. 2, row 1, which is 7.49 OD^259^ per 10^6^ cells.
*Tetrahymena thermophila*	74000000	Calzone et al.	p. 6892, Table 3,	L&M take the mean of ribosomes per cell of log phase (10.8 x10^7^) and starved cells (4.0 x10^7^).
		[43]	col. 2-3, row 4	
*Chlamydomonas reinhardtii* (chloroplast)	120500	Bourque et al [44]	p. 157, Table 2, col. 9 row 3-4	L&M take the mean of values of cytoplasmic ribosomes per hypothetical cell for the two growth conditions for wildtype cells (0.98x10^5^ and 1.43x10^5^).
*Chlamydomonas reinhardtii* (chloroplast)	55,000	Bourque et al. [44]	p. 157, Table 2, col. 10, row 3–4	L&M take the mean of the values of chloroplast ribosomes per hypothetical cell for the two growth conditions for wildtype cells (0.53 × 10^5^ and 0.57 × 10^5^).
*Ostreococcus tauri* *	1250	Henderson et al. [45]	p. 10, col. 2, l. 43	
*Adonis aestivalis* (vegetative)	47700000	Lin and Gifford [46]	p. 2481, Table 3, col. 4, row 2-4	L&M take the mean of the total number of ribosomes per cell (in millions) for the vegetative apex: central zone (40.2), peripheral zone (25.7) and rib meristem (77.2).
*Adonis aestivalis* (transitional)	39066666	Lin and Gifford [46]	p. 2481, Table 3, col. 4, row 5-7	L&M take the mean of the total number of ribosomes per cell (in millions) for the transitional apex: central zone (34.2), peripheral zone (31.1) and rib meristem (51,9).
*Adonis aestivalis* (floral)	23666666	Lin and Gifford [46]	not present	The mean of the total number of ribosomes per cell (in millions) for the floral apex is 23.9; central zone (30.9), peripheral zone (18.8), rib meristem (22.1).
Glycine max SB-1 cell	9373333	Jackson and Lark [47]	p. 236, Table 1, col. 4, row 3-5	L&M take the mean of ribosomes per cell for SB-1 cells in sucrose (9530000), M-24 cells in maltose (7190000) and M- 200 cells in sucrose (11400000); L&M exclude M-200 cells in maltose.
Rhus toxicodendron*	2400000	Vassilyev [48]	p. 620, Table 4, col. 2–4, row 6	L&M take the mean of the added numbers for cytoplasmic ribosomes per whole epithelial cell from the procambium stage (free 3260000 and bound 480000) and the SER stage (free 810000 and bound 250000).
*Zea mays* root cell	25500000	Hsiao [49]	not present	The value appears in the literature but not in Hsiao (1970a), but rather in Hsiao (1970b) p.105, Table 1, col. 4, row 4-5.
Hamster, intestinal enterocyte*	1500000	Buschmann and Manke [51, 52]	p. 23, Figure 5	In Buschmann and Manke (1981b). L&M take the mean of total ribosomes per average enterocyte for fasted and lipid-fed cells.
HeLa Cell	3300000	Duncan and Hershey [53]	p. 7229, col. 1, l. 73	Not clear how the value was calculated.
	5748830	Kulak et al. [39]	not reproducible	
	no value	Zhao [54]		
Mouse pancreas*	1340000	Dean [55]	117, Table 2, col. 6, row 12	
Rat liver cell*	12700000	Weibel et al. [56]	p. 80, Table 2, col. 10, row 12	

^1^‘ S’ refers to Supplemental Material

^2^not present - value cannot be found in the cited paper; incorrect - a wrong value was taken from the cited paper; not reproducible – calculations derived from proteomics study cannot be reproduced

^3^(Ilag et al. [57]; Gordiyenko et al. [58]; Garcia et al. [59])

*Species with both volume and ribosome count values verified

Tables 1 and 2 list on a case-by-case basis which numbers are to be found in the literature underpinning Lynch and Marinov’s Fig. 2. For the 80 values reported in Lynch and Marinov’s Appendix 1-Table 3, the most common problem we encountered is that the corresponding number does not exist (is nowhere to be found) anywhere in the paper to which it is attributed or the corresponding supplement when present. For 48 of the 80 individual values (60%) that Lynch and Marinov [1] report for cell volume vs. ribosome number, the value for the corresponding parameter is either i) not present in the cited paper ii) incorrect (a wrong value was taken from the cited paper) or iii) not reproducible (calculations derived from proteomics study that cannot be reproduced). Such cases are scored accordingly in Tables 1 and 2. All cases in which a different number is given in the cited source than that reported by Lynch and Marinov [1], are reported individually in Tables 1 and 2 for inspection. In those cases where a number was reported for the corresponding parameter, we have listed the page, the column, and the line number where the number appears.

In some cases, the numbers in Lynch and Marinov [1] are only slightly different from those reported, but the nature of those differences remains elusive. For example, Lynch and Marinov report the volume of Hela cells as 2798.668 μm^3^ (accuracy to 1/1000th of a μm^3^) citing three references, only one of which however reports the corresponding parameter, yet as 2600 μm^3^ (two significant digits). In that minority of cases where we were able to confirm the numbers reported by Lynch and Marinov [1], we have given the specific location as witness of the number’s existence.

In terms of content, Lynch and Marinov [1] distill from their analyses as their major final conclusion that their findings support the suggestion of Pittis and Gabaldon [6] that eukaryote complexity arose by piecemeal lateral gene transfer from prokaryotes (which lack the complexity they purportedly donated). It needs to be stated that the analyses of Pittis and Gabaldon [6] themselves are ridden with artifacts [7], most notably a classic case of overfitting data to a highly parameterized model (five Gaussian distributions) when a simpler model (a lognormal distribution) far better accounts for the data. Lynch and Marinov [1] also suggest that phagocytosis might have come late in eukaryotic evolution, which is hardly a novel suggestion [3, 8].

For the 26 data points presented in Fig. 2 of Lynch and Marinov, 73% are not supported by the numbers they published. The data points for one archaeon, one yeast, one unicellular alga, one plant, mouse, hamster and rat remain (marked with * in Tables 1 and 2). It is well known that ribosomes are a main constituent of prokaryotic and eukaryotic cells by weight, as cells devote about 75% of their energy budget to protein synthesis on ribosomes [4, 9].

The problem with the Lynch and Marinov paper is twofold. First, future researchers will either use those numbers from Appendix 1-Table 3 of Lynch and Marinov [1], even though they are incorrect, or worse, researchers might take the numbers presented by Lynch and Marinov and attribute them directly to the cited papers in which the numbers — in one case even the species for which the numbers are given — do not appear (Tables 1 and 2). Lynch and Marinov will thus be the point source of snowballing error through the literature.

**Fig. 1:**
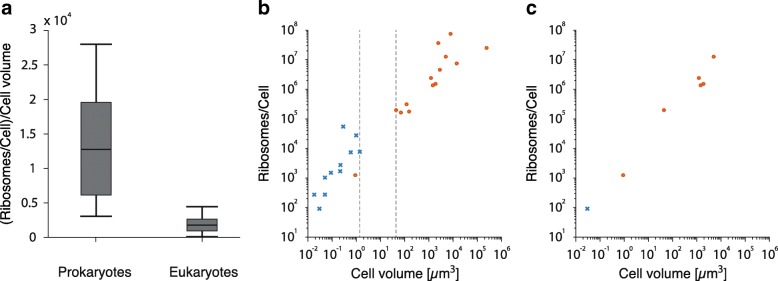
Ribosomes per cell and cell volume. **a** Boxplots of the distribution of prokaryotic and eukaryotic ‘ribosomes per cell and cell volume’-ratios. The Wilcoxon ranksum test rejected the null hypothesis that the eukaryotic and prokaryotic samples are part of the same continuous distribution with *ρ*=0.0003. **b** Ribosomes per cell versus cell volume. Blue crosses: Prokaryotes. Orange circles: Eukaryotes. Excluding *Ostreococcus tauri*, there is a gap of approximately 1.5 orders of magnitude between prokaryotic and eukaryotic cell volumes, indicating the fundamental differences in prokaryotic and eukaryotic nature. All values were taken from Lynch and Marinov [1] Appendix 1-Table 3. **c** Replot of Fig. 1b, only showing data points where both cell volume and the number of ribosomes per cell could be verified by the provided references in Lynch and Marinov [1]

Furthermore, Lynch and Marinov argue that “*the numbers of ribosomes per cell also appear to scale sublinearly with cell volume, in a continuous fashion across bacteria, unicellular eukaryotes, and cells derived from multicellular species*” [1]. In other words, Lynch and Marinov imply that there is a continuum rather than a divide between prokaryotes and eukaryotes regarding the ribosome count per cell and cell volume. However, a Wilcoxon ranksum test fails to accept the null hypothesis that the prokaryotic and eukaryotic ‘ribosomes per cell/cell volume’-ratios are both part of the same continuous distribution (Fig. 1a, *ρ*=0.0003). For this test, no values of their Appendix 1-Table 3 were altered or excluded. A simple visual inspection shows a clear divide between eukaryotic and prokaryotic data points (Fig. 1b). Excluding the smallest known autotrophic eukaryote *Ostreococcus tauri* (possessing a highly reduced genome), there is a gap of approximately 1.5 orders of magnitude between prokaryotic and eukaryotic cell volumes while ribosomes per cell only increase approximately 3-fold between representatives of the two kingdoms.

That is not the main problem, though, and our aim is not to challenge Lynch and Marinov’s inferred sublinear scaling. Rather, the point is this. Their correlations are based on values that cannot be sourced to their references, as we outline in the following. We examined 80 of the 246 literature values reported by Lynch and Marinov. More than half of the numbers we examined were modified from the literature or nonexistent there; in some cases, the numbers were the mean, sometimes the maximum from a range, sometimes the mean after some values had arbitrarily been excluded, the most common problem being that the cited literature lacked the number altogether (Tables 1 and 2). The underlying error pattern is irregular, and only seven data points remain in the plot after our inspection (Fig. 1c). But that is still not the main problem. There are 166 other values reported in Appendix 1-Tables 1 and 2 in Lynch and Marinov [1] that we did not check, because it is not our responsibility to mend the flaws in published critiques of our work with a posteriori scholarship, which should have been supplied by author-borne academic standards and rigorous peer review. Tables 1 and 2 provide no reason to trust that their other 166 numbers are any more accurate than the 80 that we inspected. Hence it is currently not possible to independently ascertain how half the numbers that Lynch and Marinov [1] ushered into print were generated and from what source they were gleaned.

This is not the first time that a paper by Lynch and Marinov has been flagged. In 2015, Lynch and Marinov [10] reported calculations for the energetic cost of a gene when in fact the issue was not about the cost of a gene (energy demands) rather about energy supply [11]. Now they argue that prokaryotes and eukaryotes represent a continuous distribution of cellular size [1] and by inference, complexity, although the converse is true (Fig. 1b). But we return to their main point, namely their claim that “*there is no reason to think membrane bioenergetics played a direct, causal role in the transition from prokaryotes to eukaryotes*”. There are indeed such reasons [2], it is worthwhile to recapitulate them briefly.

The issue is the evolutionary origin of cellular complexity [4]. Mitochondrial respiration was present in the eukaryote common ancestor [2–4]. Why? Eukaryotes and prokaryotes generate the same amount of energy per unit volume [4]. In prokaryotes, chemiosmotic energy conservation occurs at the plasma membrane, in eukaryotes it occurs in mitochondria, which however constitute only about 10% of the cell volume [12]. That is why eukaryotic cell size is not evolutionarily constrained, while prokaryotic cell size is [2, 4]. It is also why human mitochondria operate at about 50 °C [13], they provide the energy for the remaining 90% of the cell, which is cytosol [2] consisting of 400 mg/ml protein. Mitochondria afford eukaryotes the ability to explore the (over)expression of novel proteins in that cytosol, an option that prokaryotes do not have [2].

## Conclusion

Lynch and Marinov might wish to follow our example of providing the page, column, and line, for the position of all 246 numbers that they attribute to the literature and explain how each value was determined from which selected range of numbers (and excluding which) in the original literature they cite. For a sample of one third of the values underpinning the paper by Lynch and Marinov [1], more than 50% of the numbers fail independent inspection, are not to be found in the cited literature, and hence cannot have been checked by either author prior to publication. The source of their numbers is obscure. This paper was originally submitted to eLife and rejected. During revision of this paper, a correction of the original paper by Lynch and Marinov [1] was published. The original version of their paper (including Appendix 1-table 3, Figure 2 and its caption) is no longer available at eLife, but can still be obtained here: http://www.molevol.de/LM2017.pdf.

## Methods

MG obtained the papers cited by Lynch and Marinov [1] that underlie their Fig. 2 and read the papers in search of the values. Her results were spot checked by WFM, then each number was thoroughly checked, one by one, first by MK and JX, then again by NK and JX. The results of that fact checking were scored in Tables 1 and 2, where it was recorded whether the numbers presented by Lynch and Marinov could be confirmed (stating page, column, and line), whether different numbers for the corresponding parameter or a range were presented (stating page, column, and line), or whether the number was not present in the paper cited. For the Wilcoxon ranksum test, all values were collected from Appendix 1-Table 3 of Lynch and Marinov [1]. No values have been excluded nor altered in any way.

## Reviewers’ comments

### Reviewer’s report 1: Eric Bapteste, CNRS, Université Pierre et Marie Curie, Paris, France

**Reviewer comment:** Debates about theories and about evidence backing up these theories are the norm (and, as a pluralist, I would even say are welcome) in any active research field. If anything, they keep a field alive, and stimulate original thoughts. Hence, I sincerely admire both Bill Martin’s and Michael Lynch’s numerous inspiring contributions to evolutionary biology. Martin’s present article precisely questions the validity of the quantitative evidence used by Lynch and Marinov (hinting at the repeated use of averaged values, and reporting several values without clear bibliographical origins). I agree that this situation is frustrating, and I think that, ideally, doubts raised about some of these numbers should be explicitely addressed. I suggest that a journal like Biology Direct could be a good venue to publish an updated version of the incriminated tables, in a way that clarifies the origins of some of the evidence used by Lynch and Marinov, and, as an avid reader of both Martin’s and Lynch’s works, I would even hope that these updated numbers could then be used to update computations about energetics, to determine whether such (carefully checked) numbers do or do not impact Lynch and Marinov’s former conclusions.

**Author’s response:** *We thank R1 for the endorsement of our paper. We share the expectation to see a corrected version of the tables by Lynch and Marinov, clearly sourced (including, as we did here, page, column and row of the cited source for the given values).*

**Reviewer comment:** The criticisms on Pittis and Gabaldon’s article also seem somehow out of place in the present MS.

**Author’s response:** *We must politely disagree in this regard. We believe it is well-worth noting that a common trend seems to be emerging in the community, a trend of misuse of statistics in extrapolations on complex biological questions. We hope that our criticism can point to the importance of both i) the collection of more well-sourced, quality data and ii) a pondered use of statistics as a tool, not as an end, in biology.*

### Reviewer’s report 2: Jianzhi Zhang, EEB, University of Michigan, MI, U.S.A.

**Reviewer comment:** There is an ongoing debate on the role of energy production by mitochondria in the origin of eukaryotes and their subsequent diversification. In 2017, Lynch and Marinov published an article in eLife suggesting that eukaryotes are energetically no more efficient than prokaryotes. Because their empirical evidence is based on the analysis of various data from the literature, the accuracy of these data is critical to their conclusion. In the present manuscript, Gerlitz et al. systematically examined the sources of the data used by Lynch and Marinov. They report that most of the data cannot be found in the references where the data were claimed to be from or were different from the values in these references. Although the causes of these discrepancies are unclear, it is important to alert the research community the potential invalidity of Lynch and Marinov’s conclusion by publishing these discrepancies. I must say that I do not believe that reviewers are responsible for the accuracy of the data in a paper, so I have not closely examined the numbers in the two tables of the present manuscript. While Lynch and Marinov should be responsible for the accuracy of their paper, Gerlitz and colleagues bear the responsibility for this manuscript. My two major comments and a minor comment follow.

**Author’s response:** *We thank R2 for sharing our concern with the importance of the accuracy of published data. We hereby confirm that we bear full responsibility for the content of this manuscript.*

**Reviewer comment:** Major: 1. Because a large fraction of the data in Lynch and Marinov cannot be verified by Gerlitz et al., I wonder if the general trends reported by Lynch and Marinov also disappear. Specifically, it will be interesting to replot the two figures in Lynch and Marinov using the verified portion of the data. I understand that whether the data used are sound and whether the results are robust are different issues, and I think Gerlitz et al. can address both issues in this work.

**Author’s response:** *This is a good point to which we have given considerable thought. The problem is that the values that stand upon our close inspection are so few as to produce an almost empty plot. Only one prokaryotic data point survives the inspection. We believe that the correction of the values needs to be provided by Lynch and Marinov, either as a corrigendum or in a new paper, it is not our duty to correct their paper. We intend here to alert the community to the inaccuracy of the data provided by Lynch and Marinov. We would also like to see more data points added, rather than just a handful of species, in order to sustain such bold claims.*

*Nonetheless, for fairness we provide an additional plot showing the data points that were accurately attributed in Lynch and Marinov 2017 as* Fig. 1c*, it clearly does not impinge upon our observation that the values for prokaryotic and the eukaryotic cells plotted by Lynch and Marinov 2017 are drawn from distinct distributions.*

**Reviewer comment:** 2. Gerlitz et al. made a point in Fig. 1 that prokaryotes and eukaryotes differ by 1.5 orders of magnitude and showed a significant *p*-value from a Wilcoxon ranksum test. If my understanding is correct, this test result indicates that ribosomes concentration (# of ribosomes/cell/cell volume) is lower in eukaryotes than in prokaryotes. However, this is not what Lynch and Marinov argued about in the corresponding Fig. 2 of their paper. Lynch and Marinov’s Fig. 2 argues that the general scaling relationship between # of ribosome per cell and cell volume is not different between prokaryotes and eukaryotes. Only when the scaling factor is 1 will ribosome concentration be constant, and the scaling factor is 0.79 according to Lynch and Marinov. In other words, Lynch and Marinov and Gerlitz et al. do not disagree here.

**Author’s response:** *This is a fair point. However, we wish only to show, that contrary to Lynch and Marinov’s claims, even when using their (cryptic) data, the power law they describe does not come from a continuous distribution. The issue of continuity is key. As they say: “[…] the numbers of ribosomes per cell also appear to scale sublinearly with cell volume,* ***in a continuous fashion across bacteria, unicellular eukaryotes, and cells derived from multicellular species*** *”. They state it again later “The numbers of both ribosomes and ATP synthase complexes per cell, which jointly serve as indicators of a cell’s capacity to convert energy into biomass, scale with cell size* ***in a continuous fashion both within and between*** *bacterial and eukaryotic groups”. Both of our figures show that this is not the case. This criticism brings out a very good point, actually. We return to this point later in a reply to Martin Lercher. The issue of continuity is now addressed more explicitly in the revised text to underscore this important point.*

### Reviewer’s report 3: Martin Lercher, Heinrich-Heine-Universität Düsseldorf, Germany

**Reviewer comment:** Gerlitz et al. checked the values (cell volume and ribosome counts) underlying Fig. 2 in Lynch & Marinov (2017, abbreviated LM below) against the reported literature sources. They could not find a large fraction of the reported cell volume values, while many of the ribosome counts were calculated as averages over sets of reported values according to an undisclosed (or non-existent) algorithm by Lynch & Marinov. Gerlitz et al. conclude that the data in Lynch & Marinov (2017) does not conform to scientific standards. After carefully going through the problems with the LM data reported by Gerlitz et al., I have to agree that the standards of data collection employed by LM do not conform to current scientific standards. While it is not clear to what extent the data is factually wrong (within some acceptable margin of error), the lack of a clear standard according to which LM obtained those data is reason for concern. No experimental work that employed similarly loose algorithms to arrive at data values would (or should) be considered for publication in a peerreviewed journal.

**Author’s response:** *We thank R3 for sharing our concerns with the quality standards of published data and analysis.*

**Reviewer comment:** p.7 l.164: “a Wilcoxon ranksum test fails to accept the null hypothesis that the prokaryotic and eukaryotic ‘ribosomes per cell/cell volume’-ratios are both part of the same continuous distribution (Fig. 1a …).” That prokaryotic and eukaryotic ‘ribosomes per cell/cell volume’-ratios are both part of the same continuous distribution is not what was stated by LM. Instead, they state that a power-law relationship can be fitted successfully to the ribosome count/cell versus cell size data. Thus, Fig. 1a and the Wilcoxon test do not address a hypothesis posited by LM, and I would recommend removing both.

**Author’s response:** *As summarized in our reply to Referee 2, Lynch and Marinov do state in the Results and Discussion sections of their paper, “[…] the numbers of ribosomes per cell also appear to scale sublinearly with cell volume,* ***in a continuous fashion across bacteria, unicellular eukaryotes, and cells derived from multicellular species*** *(Fig. 2)” and that “The numbers of both ribosomes and ATP synthase complexes per cell […] scale with cell size* ***in a continuous fashion both within and between*** *bacterial and eukaryotic groups”. What we see in their (cryptic) data is not a continuous scaling, but a clear divide between the average concentration of ribosomes of prokaryotic and eukaryotic cells.*

**Reviewer comment:** p.7 l.171: “there is a gap of approximately 1.5 orders of magnitude between prokaryotic and eukaryotic cell volumes while ribosomes per cell only increase approximately 3-fold between representatives of the two kingdoms”. Another, more rigorous way of looking at this issue would be to fit two independent power laws (linear fits on log-log scale) to the prokaryotic and to the eukaryotic data, and to see if (a) these two power laws are significantly different, and (b) if the data is more appropriately described by two rather than one power law.

**Author’s response:** *We agree that it would be interesting to look at two power laws independently. However, for that to be done accurately we would require the correct, well-sourced values that we show that Lynch and Marinov failed to provide. We believe it is not our duty to correct the values of Lynch and Marinov. We look forward to seeing them providing such analyses, and if possible more data points than a handful of prokaryotes and eukaryotes, to make such bold claims. We agree that the analysis that Referee 3 suggests would be a much better approach for sustaining such conclusions. There is a danger in just comparing R* ^*2*^ *values of the three power-laws though, but we believe this is not the place to advise Lynch and Marinov about which methods they should use in future papers.*

**Reviewer comment:** Table 2. Many of the values reported as “not present” by the authors are – as inverse engineered by the authors and stated in the comments – mean values of proteomic estimates for different ribosomal subunits. This was actually stated by LM in the header of appendix1-Table 3: “proteomic estimates are from averaging of cell-specific estimates for each ribosomal protein subunit”, so I would consider the label “not present” as inappropriate (even if the exact calculations are frequently incorrect, as pointed out in the comments in Table 2).

**Author’s response:** *This is a good point. We used “not present” as a standard tag for values that could not be found in the cited reference, were incorrect, or could not be reproduced properly. We now use all three, more specific tags: “not present”, “incorrect” and “not reproducible”.*

**Reviewer comment:** I have only one remaining concern. It relates to the statement by Lynch and Marinov: “the numbers of ribosomes per cell also appear to scale sublinearly with cell volume, in a continuous fashion across bacteria, unicellular eukaryotes, and cells derived from multicellular species”. The choice of the word “continuous” here is unfortunate; it probably refers to the observation that a line drawn through the data in A can be continued through the data in B. Let us define x:=“cell volume”, y:=“the number of ribosomes per cell”. What Gerlitz et al. test (l.170ff) is the null hypothesis that y/x for data in bacteria and in eukaryotes come from the same distribution. This would be appropriate if Lynch and Marinov had observed a linear scaling, y = c*x. However, Lynch and Marinov explicitly state that there is a sub-linear scaling: in the legend to Fig. 2, they give the inferred relationship as y = 8551*x^0.79. Thus, to show disagreement with Lynch and Marinov, Gerlitz et al. would have to compare the distributions of y/(x^0.79) instead of comparing the distributions of y/x between bacteria and eukaryotes.

**Author’s response:** *The point is well taken and has been addressed in the main text.*

## Abbreviations

ATPase: Adenosin triphosphatase
OD: Optical density
